# Sialadenoma papilliferum of the tongue mimicking a malignant tumor

**DOI:** 10.5935/1808-8694.20130071

**Published:** 2015-10-04

**Authors:** Jean Nunes dos Santos, Adna Conceição Barros, Clarissa Araújo Gurgel, Luciana Maria Pedreira Ramalho

**Affiliations:** a PhD in Oral Pathology (Associate Professor).; b MSc in Odontology at the Federal University of Bahia (PhD student, Odontology, Federal University of Bahia).; c PhD in Pathology (Adjunct Professor at the Federal University of Bahia).; d PhD in Stomatology (Associate Professor at the Federal University of Bahia). Federal University of Bahia. Surgical Pathology Lab - Dentistry School.

**Keywords:** mouth, salivary gland neoplasms, tongue neoplasms

## INTRODUCTION

Sialadenoma papilliferum is a rare tumor of the salivary gland described for the first time by Abrams & Finck in 1969^1^. It is histologically similar to syringocystadenoma papilliferum^2^. The origin of this tumor is yet unclear, although reports indicate it appears to stem from myoepithelial cells^1^, blocked glandular ducts resulting in hyperplasia^3^, metaplastic phenomena^4^, intercalated duct cells^5^, or excretory duct cells^6^.

Sialadenoma papilliferum often involves the palate^6^, and only one case has been reported to date in Portuguese^2^. This case report discusses the clinical and pathological findings of a patient with minor salivary gland sialadenoma papilliferum of the tongue.

## CASE REPORT

**Figure 1:**
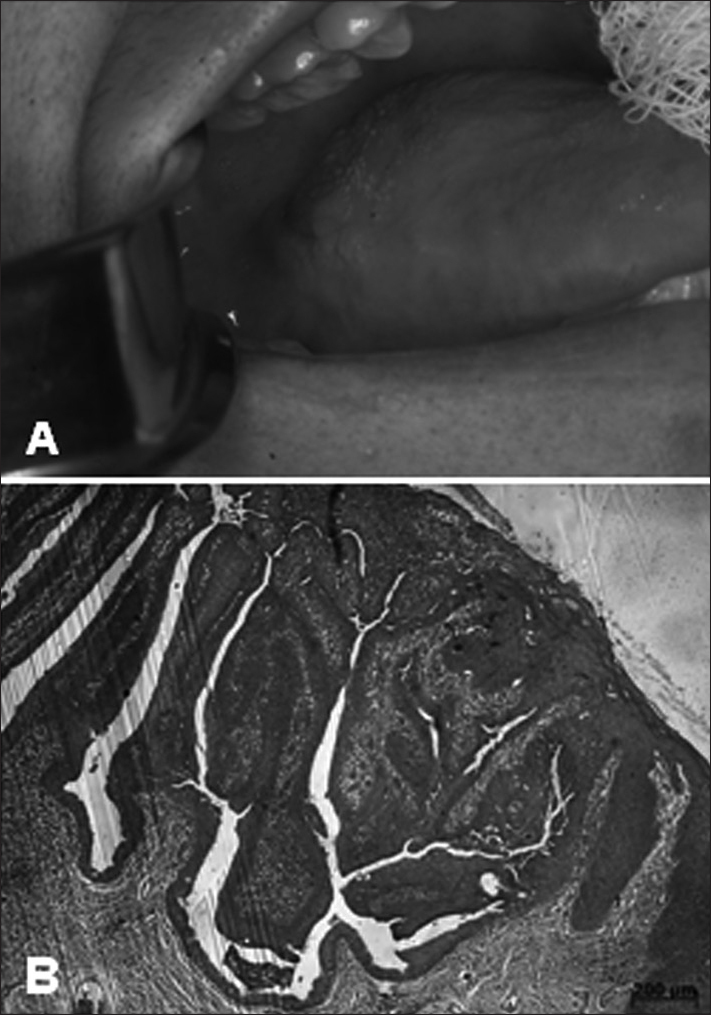
A: Irregular tumor located in the right lateral border of the tongue. B: Endophytic tumor with papilliferous squamous epithelium surface; the base of the tumor presents a transition from squamous epithelium to ductal columnar epithelium.

A 32-year-old brown female patient came to the stomatology clinic complaining of a painless lump in the back of her tongue that had been evolving for a year. Physical examination showed an exophytic irregular lesion, with a discretely papillomatous surface located in the right posterior lateral border of the tongue measuring approximately 1.0 x 1.0 cm (Figure 1A). The patient said she did not smoke or drink alcohol, and in neck palpation no suspicious nodes were felt. Incisional biopsy was done as she was suspected to have a squamous cell carcinoma. Pathology tests showed presence of superficial ulcerations on the mucosa covered with parakeratinized stratified squamous epithelium parallel to endophytic growth of papilliferous squamous epithelium supported by connective tissue with significant inflammation. The base of the tumor presented a transition from papilliferous squamous epithelium to columnar duct epithelium covering proliferating ductal elements, which had cuboidal basal cells and overlying columnar cells, in addition to mucocytes (Figure 1B).

Ductal, at times branching, elements and small cystic spaces at the depth of the tumor were present. Histopathology diagnosis was consistent with sialadenoma papilliferum of the tongue. Despite the scarcity of material, immunohistochemistry tests were run using cytokeratins 7, 13, and 14 applied in an polymer amplification system (Envision™, Dako Cytomation). Cytokine 13 was strongly labeled. The tumor was completely removed, although the biopsy report described partial removal of the lesion. The patient has been followed for almost two years without signs of recurrent disease.

## DISCUSSION

The patient's tumor involved the lateral posterior border of the tongue. Sialadenoma papilliferum in this location has not been reported in the literature, except for one case described by Liu et al.^2^ from which arose a mucoepidermoid carcinoma. Therefore, this is a quite rare site for oral involvement, once the palate is the preferential location of this tumor type^6^.

In clinical terms, the tumor had an irregular, discretely papillomatous surface, which led to the consideration of squamous cell carcinoma. However, histology tests ruled out this suspicion, despite the squamous differentiation and papilliferous aspect of the tumor, at times seen in squamous cell carcinomas. Our patient had a tumor with endophytic growth, papilliferous squamous epithelium, with visible transition between papilliferous epithelium and proliferative ductal epithelium with cuboidal cells, columnar cells and mucocytes. Therefore, the morphological aspects described were consistent with sialadenoma papilliferum^1^, ^2^, ^5^, ^6^. It is worth remembering that exophytic growth^2^, ^6^ and inflammation^2^, ^6^ have also been associated with sialadenoma papilliferum. Immunohistochemistry findings were consistent with the origin of the tumor being the glandular excretory ducts, as confirmed by tests labeled for CK13^6^. This is why care must be taken in the differential diagnosis against mucoepidermoid carcinoma and ductal papilloma.

The patient's tumor was completely removed, and she has been free of disease for almost two years without signs of recurrence, as similarly reported by Liu et al.^2^.

## CLOSING REMARKS

The diagnosis of sialadenoma papilliferum requires careful examination, as tumors of the tongue may be malignant, and benign tongue tumors of the tongue may mimic malignant disease.
